# Photocatalytic Degradation of Dyes Using Titania Nanoparticles Supported in Metal-Organic Materials Based on Iron

**DOI:** 10.3390/molecules27207078

**Published:** 2022-10-20

**Authors:** Elizabeth Rojas-García, Diana Carolina García-Martínez, Ricardo López-Medina, Fernando Rubio-Marcos, Aldo A. Castañeda-Ramírez, Ana M. Maubert-Franco

**Affiliations:** 1 Área de Ingeniería Química, Departamento de Ingeniería de Procesos e Hidráulica, Universidad Autónoma Metropolitana-Iztapalapa, Mexico City 09340, Mexico; 2 Área de Química de Materiales, Departamento de Ciencias Básicas e Ingeniería, Universidad Autónoma Metropolitana-Unidad-Azcapotzalco, Mexico City 02200, Mexico; 3 Área de Procesos de la Industria Química, Departamento de Energía, Universidad Autónoma Metropolitana-Unidad Azcapotzalco, Mexico City 02200, Mexico; 4 Electroceramic Department, Instituto de Cerámica y Vidrio, CSIC, Kelsen 5, 28049 Madrid, Spain; 5 Escuela Politécnica Superior, Universidad Antonio de Nebrija, C/Pirineos, 55, 28040 Madrid, Spain

**Keywords:** MIL-100 (Fe), TiO_2_/MIL-100 composite material, Orange II, Reactive Black 5, dye photocatalytic degradation, MOFs

## Abstract

Composite materials based on titania nanoparticles (TiO_2_ NPs) and three metal-organic frameworks (MOFs) called *MIL-53 (Fe)* ((Fe (III) (OH) (1,4-BDC)), MILs (Materials Institute Lavoisier)), *MIL-100 (Fe)* (Fe_3_O(H_2_O)_2_OH(BTC)_2_), and *Fe-BTC* (iron-benzenetricarboxylate) with different percentages of TiO_2_ NPs (0.5, 1, and 2.5% wt.) were synthesized using the solvothermal method and used as photocatalytic materials in the degradation of two dyes (Orange II and Reactive Black 5 (RB5)). The pristine and composite materials were characterized with X-ray diffraction, Raman, UV–Vis and Fourier transform infrared spectroscopy and scanning electron microscopy techniques. The 2.5TiO_2_/MIL-100 composite material showed the best results for the degradation of both dyes (Reactive Black 5 and Orange II dye, 99% and 99.5% degradation in 105 and 150 min, respectively). The incorporation of TiO_2_ NPs into MOFs can decrease the recombination of the change carrier in the MOF, increasing the photocatalytic activity of a pristine MOF. Results therefore indicated that the synthesized MOF nanocomposites have good potential for wastewater treatment.

## 1. Introduction

The liquid water essential for life on our planet is at serious risk due to pollution caused by anthropogenic activities and industrialization that have grown since the middle of the last century. The availability of freshwater controls most of the world’s economy. One-sixth of the world’s population suffers from limited access to freshwater [1]. Polluted waters cause health diseases and damage to ecosystems due to various pollutants such as biological, inorganic, and organic. Dyes are organic compounds exhibiting a worrying increase in prevalence in the environment. Different methods are used to eliminate dyes, such as physical methods (adsorption, the most used process) with a wide variety of adsorbent materials, from low cost techniques to the most innovative method currently [2,3], or chemical methods such as advanced oxidation, membrane filtration, enzymatic treatment, sonication, and photocatalysis [4,5,6].

The photosensitization effect reported by Fujishima and Honda [7], who found that the electrolysis of H_2_O proceeded at a much lower voltage as compared to normal electrolysis using a TiO_2_ photoanode irradiated with UV light, is currently applied in various reactions, particularly the photodegradation of organic dyes by catalysts in aqueous/dispersion systems by UV irradiation due to its environmentally friendly method, low cost, and lack of secondary pollution [8,9,10,11,12,13,14]. Among other solids (ZnO, Fe_2_O_3_, CdS, GaP, and ZnS), the most widely used semiconductor is the anatase crystalline form of titanium dioxide (TiO_2_), since in addition to being the most active for photocatalysis, it is a relatively cheap material, chemically and biologically inert, and resistant to photo-corrosion. The organic pollutants are thus oxidized via direct hole transfer or, in most cases, attacked by the *OH radical formed in the irradiated TiO_2_ [15,16]. Furthermore, a new class of organic–inorganic materials called metal-organic frameworks (MOFs) or porous coordination polymers (PCPs) has emerged. Metal-organic frameworks (MOFs) or porous coordination polymers (PCPs) are hybrid organic–inorganic porous materials consisting of organic linkers and metal-oxo clusters. MOFs are solid crystalline hybrid materials formed by a network of metal ions linked to multidentate organic molecules through coordination bonds. Their various applications as adsorbents, separation materials, ion conductive materials, or catalysts are active research areas. The first materials of this type were described in the middle of the last century. However, it was not until the 1990s that the groups of Li et al. [17], Barton et al. [18], Riou et al. [19], Loiseau et al. [20], Livage et al. [21], Simon et al. [22], Serre et al. [23], Sasoye et al. [24], Kitagawa et al. [25], and Jhung et al. [26] promoted this field of research. Numerous methods of synthesis of MOFs have been described [27]. Solvothermal crystallization is the most used. Our research group is intensely interested in synthesizing MOFs in various applications, particularly photocatalysis [28].

**Figure 1:**
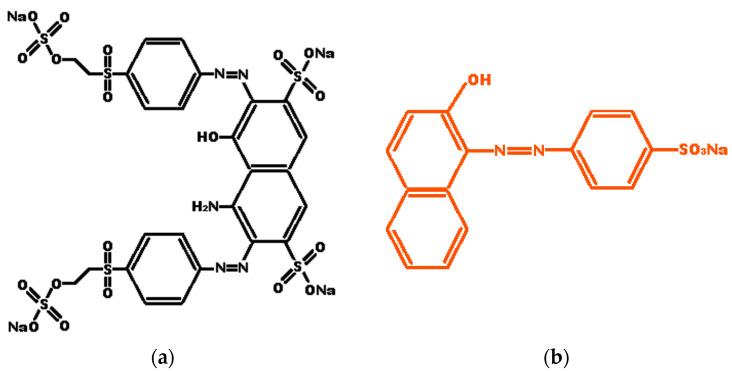
(**a**) Chemical structure of Reactive Black 5 (NR5) dye; (**b**) Chemical structure of Orange II dye.

Reactive Black 5 (RB5) (Tetrasodium-4-ammonium-5-hydroxy-3.6 (Bis4-(2(Sulfonatoox) Ethylsulfonyl)Phenyl)Azo)-Naphthalene-2,7-disulfonate) is a di-azo sulfonic (Figure 1), composed of an auxochromic–chromophore complex made up of the azo group and aromatic rings attached to it. This interaction determines the amount of light absorbed and the intensity of the color. On the other hand, the dye has a sodium group (+NaSO_3_) in its structure, which determines the compound’s solubility. RB5 is commonly used in the textile industry. It presents a high level of environmental contamination because more than 15% of its content goes directly to wastewater and is highly toxic [29,30]. The azo dye Orange II, as shown in Figure 1, represents more than 15% of the world’s dyes used in the textile manufacturing industry. Orange II (C1_6_H_11_N_2_NaO_4_S, 4-(2-Hydroxy-1-naphthyl azo) benzenesulfonic acid sodium salt is an anionic surfactant mono-azo textile dye of the acid class. It is resistant to light, O_2_ action, and degradation by common acids or bases. In wastewater treatment plants, Orange II does not undergo biological degradation. The high stability of Orange II is helpful in textile manufacturing, but a problem arises later due to difficulty managing its removal [31].

This work is focused on the degradation of two industrially important synthetic dyes, namely Reactive Black 5 (RB5) and Orange II (Figure 1), with photocatalysis employing pristine MOFs and MOFs modified by TiO_2_ nanoparticles. Fe-based MOFs Fe-BTC, MIL-53 (Fe), and MIL-100 (Fe) were synthesized using solvothermal methods. Furthermore, they were incorporated into each MOF synthesized with small quantities of TiO_2_ nanoparticles (0.5, 1.0, and 2.5%). The measurement of the degradation of the dye was carried out employing visible radiation.

## 2. Results and Discussion

### 2.1. Material Characterization

#### 2.1.1. X-ray Diffraction

Figure S1 shows the powder X-ray diffraction pattern (PXRD) of the TiO_2_ NPs. Several peaks are observed at 2θ = 25, 37, 48, 54, 55, 62, 71, and 75° corresponding to the (101), (112), (200), (105), (211) (204), (220), and (215) crystallographic planes, respectively, which are characteristics of the anatase phase of TiO_2_ [32,33]. The crystallite size calculated from Sherrer’s equation using the highest intensity peak at 2θ = 25° corresponding to the (101) plane was 43.1 nm, classified as a nanostructured material.

**Figure 2:**
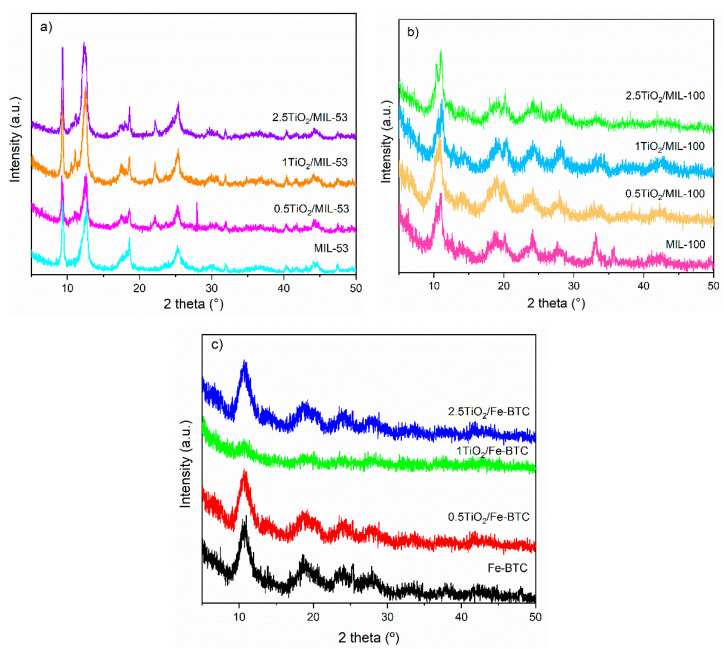
PXRD of (**a**) MIL-53 (Fe) series, (**b**) MIL-100 (Fe) series, and (**c**) Fe-BTC series.

Figure 2a shows the powder X-ray diffraction patterns of the MOF MIL-53 (Fe) and supported materials. In the PXRD patterns of MIL-53 (Fe) and composite materials, diffraction peaks at 2θ = 9.3, 12.3, 18.6, 22.1, 25.3, and 31.3 ° are observed, characteristics of MOF MIL-53 (Fe), according to the literature [33]. This MOF comprises Fe (III) cations and organic linker (1,4-dicarboxylic acid), creating a three-dimensional framework with a one-dimensional pore channel system. The incorporation of TiO_2_ NPs into MOF MIL-53 (Fe) does not show significant changes in its structure. Composite material PXRD patterns do not show diffraction peaks corresponding to TiO_2_, probably due to the small quantities of TiO_2_ NPs incorporated into the MOF.

Figure 2b shows the X-ray diffraction patterns corresponding to the MOF MIL-100 (Fe) and those incorporated with the TiO_2_ NPs. In all PXRD patterns, peaks are observed corresponding to MOF MIL-100 (Fe), according to literature, which is a three-dimensional crystalline material prepared from a combination of Fe (III) salt and the tricarboxylate ligand (trimesic acid). This material has been reported as a highly porous MOF. The two mesoporous cage sets (24 and 29 Å) are reportedly accessible through the microporous windows (ca. 8.6 and 4.7–5.5 Å) of the material [34,35]. The most intense peak at 2θ = 10.9° was used to calculate the crystallite size with the Scherrer equation. All materials showed a crystallite size of 7.2 nm. The incorporation of TiO_2_ NPs into MOF MIL-100 (Fe) does not show significant changes in its structure. In this case, composite material PXRD patterns do not show diffraction peaks corresponding to TiO_2_ NPs, due probably to the small quantity of TiO_2_ NPs incorporated into the MOF.

Figure 2c shows the power X-ray diffraction patterns of the MOF Fe-BTC and materials incorporated with the TiO_2_ NPs. As can be observed, the Fe-BTC PXRD pattern shows diffraction peaks at 2θ = 10.8, 14.3, 18.7, and 24.1°, which are characteristic of these materials, with the highest peak at 10.8° [36]. This MOF shows broad diffraction peaks indicating its semi-amorphous nature, with small crystal size and disordered structure. Those same peaks are observed in the materials incorporated with different percentages of TiO_2_ NPs. Remarkably, the MOF Fe-BTC structure was not altered after incorporating the TiO_2_ NPs in the materials with compositions of 0.5 and 2.5% wt.

#### 2.1.2. Raman Spectroscopy

**Figure 3:**
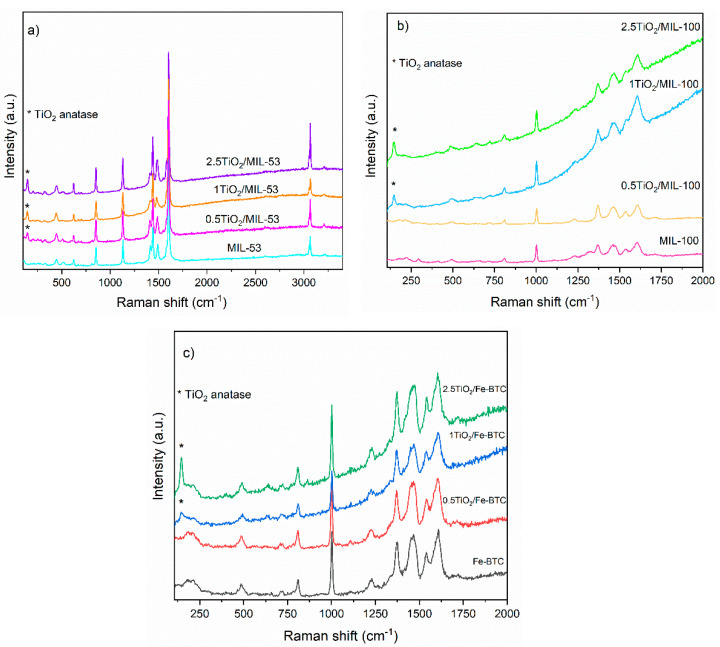
Raman spectra of (**a**) MIL-53 (Fe) series, (**b**) MIL-100 (Fe) series, and (**c**) Fe-BTC series.

Figure 3a presents the Raman spectra of the MOF MIL-53 (Fe) and TiO_2_/MIL-53 (Fe) composite materials. The region between 1800 and 860 cm^−1^ is dominated by the vibration modes of the organic ligand (terephthalic acid) and the interaction between the metal and BDC organic ligand. Several bands are observed in the region between 1595 and 1347 cm^−1^, corresponding to an interaction between the metal ions and BDC organic ligand. TiO_2_/MIL-53 (Fe) composite materials show similar Raman spectra and maintain the leading bands of MIL-53 (Fe). However, there are some differences, and a new band appears at 142 cm^−1^. This band is characteristic of the presence of the TiO_2_ anatase phase.

Raman spectra for MOFs with organic BTC ligand (MIL-100 (Fe) and Fe-BTC), for the pristine MOFs and supported materials, are shown in Figure 3b,c.

The Raman spectra of both series can be divided into two regions. The first region observed is above 740 cm^−1^, associated with the organic ligand, where C-H bond vibrations (between 826 and 762 cm^−1^) and C=C bonds (1612 and 1003 cm^−1^) of the benzene ring and the interactions of the carboxylate groups (1546 and 1461 cm^−1^) are observed. The second region is below 600 cm^−1^, where it is possible to keep the interaction of the metal (Fe) coordinated with the oxygen of the organic part. The incorporation of TiO_2_ NPs is evidenced by differences observed between the Raman spectrum of the Fe-BTC MOF (without NPs) and the MOFs with TiO_2_ NPs. The signal at 142 cm^−1^ refers to the presence of the TiO_2_ anatase phase, which evidences its incorporation.

#### 2.1.3. Scanning Electronic Microscopy (SEM)

**Figure 4:**
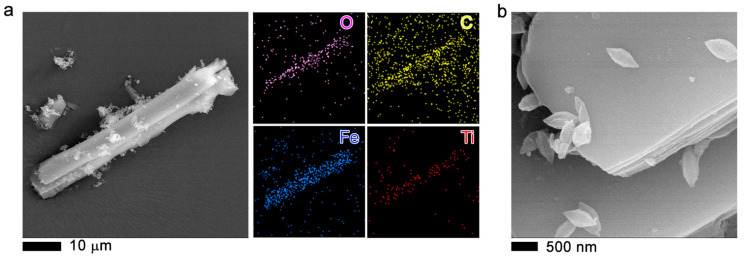
SEM images (**a**,**b**) of 2.5TiO_2_/MIL-53 (Fe) composite material. Additionally, the elemental energy dispersive X-ray spectroscopy (EDS) maps are plotted on the right of the panel (**a**).

**Figure 5:**
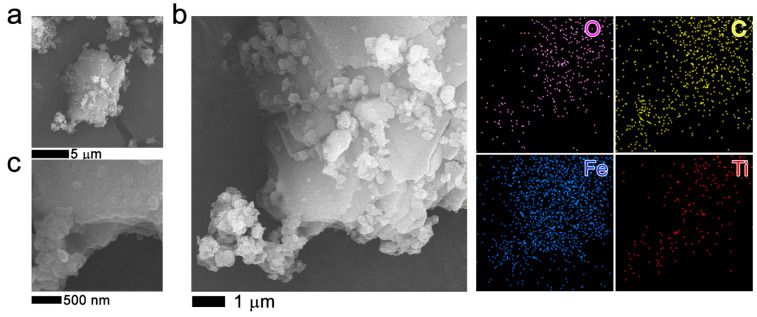
SEM images (**a**–**c**) of 2.5TiO_2_/MIL-100 (Fe) sample. The elemental analysis with energy dispersive X-ray spectroscopy (EDS) is plotted on the right of the SEM image.

Figure 4a,b show the 2.5TiO_2_/MOF MIL-53 (Fe) morphology. It is observed that the structure of MOF MIL-53 (Fe) is mainly made up of elongated crystals with an acicular habit-forming laminar bar (see Figure 5a,b). In addition, small, flat, diamond-shaped structures are observed that could give rise to bars (Figure 4a). The sample mapping obtained by X-ray mapping shows areas of higher density of points corresponding to a higher concentration of the element, in this case Fe and C (corresponding to the support of the sample) [37]. A homogeneous distribution of Ti atoms was also observed.

Figure 5a–c show the SEM images of 2.5TiO_2_/MOF MIL-100 (Fe) and elemental analysis with energy dispersive X-ray spectroscopy (EDS). Those images show agglomerations consisting of nonhomogeneous particles (Figure 5a) formed by layers (Figure 5a,c), thus creating the characteristics of these very porous materials. EDS mapping of the elements of Fe, O, C, and Ti shows a homogeneous distribution.

**Figure 6:**
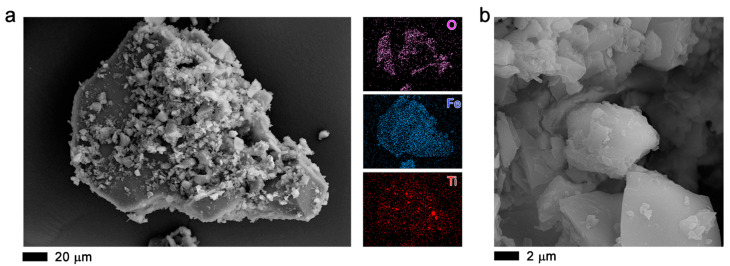
MOF Fe-BTC, SEM images (**a**,**b**). The elemental analysis with energy dispersive X-ray spectroscopy (EDS) is plotted on the right of panel (**b**).

In the scanning electron microscopy images, the formation of agglomerates (Figure 6a,b) is observed, similar to the commercial Basolite F300, which is semi-amorphous. There is the formation of platelets or sheets, which layer the crystal, making the material porous. The material is made up of the organic and metal parts. Elemental analysis shows the presence of Fe, and the other elements are part of the sample holder and the sample coating. This same morphology is observed in the supporting materials, indicating that the incorporation of TiO_2_ NPs into the MOF does not show a change in the morphology of the MOF (Figure S1).

#### 2.1.4. Optical Measurements

The samples’ bandgap energy (Eg) was determined by transforming the diffuse reflectance UV–Vis spectra treated with the Tauc equation [38]. A tangent line to the slope is generated in an electronic transition (Figure S2) to determine the solids’ direct and indirect bandgap. The Eg values for all the materials synthetized are shown in Table 1. As can be observed, all materials synthetized could be activated with visible light. The incorporation of TiO_2_ NPs into the MOF slightly decreases the bandgap. MOF MIL-100 and composite materials show a smaller bandgap. The bandgap reduction helps degrade dyes, which is essential if the photoexcited electrons can be transferred effectively from the MOF to the semiconductor.

**Figure 7:**
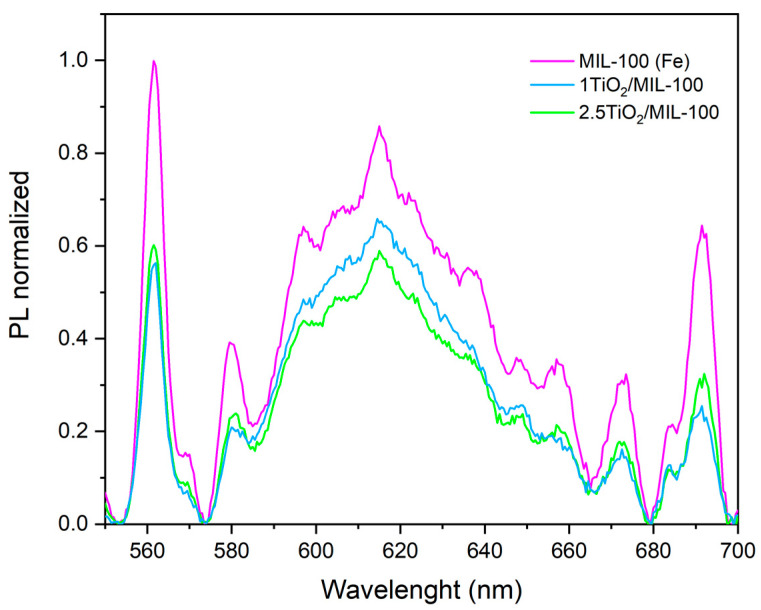
PL excitation spectra of MIL-101 (Fe) and supported materials.

The PL spectra (Figure 7) show that 2.5TiO_2_/MIL-100 composite material has a low PL emission, while MIL-100 (Fe) has a high PL peak intensity, indicating that 2.5TiO_2_/MIL-100 has a faster charge transfer ability with a prolonged lifetime of electron–hole pairs. This behavior could explain the greater activity that this material presents. The introduction of TiO_2_ in the MIL-100 (Fe) can separate the electron–hole pairs, reducing their recombination. The decrease in the recombination of charge carriers could increase the photocatalytic activity in the photocatalytic degradation of Orange II and Reactive Black 5 dyes as was observed previously.

### 2.2. Photocatalytic Activities

#### 2.2.1. Photocatalytic Degradation of Orange II Dye

**Figure 8:**
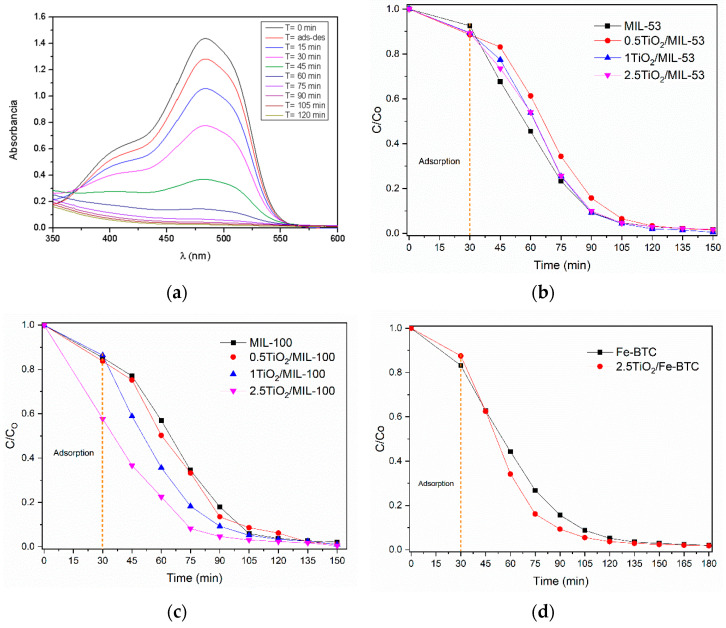
Photocatalytic activity of the Orange II dye for the different materials: (**a**) UV–Vis spectra obtained of the Orange II photocatalytic degradation using the 2.5TiO_2_/MIL-100 sample; (**b**–**d**) C(t)/C_0_ concentration ratio vs. reaction time for MIL-53 (Fe), MIL-100 (Fe) and Fe-BTC, respectively.

Figure 8a shows the UV–Vis spectra obtained of the Orange II photocatalytic degradation using the 2.5TiO_2_/MIL-100 sample. In this case, a strong absorption band is observed at 485 nm corresponding to the N=N chromophore group, this being the characteristic peak used to follow the kinetics of the reaction. It shows a gradual decrease of absorbance as the degradation time increases; after 105 min of being exposed to light, it almost eliminates the dye. Figure 8b–d show the C(t)/C_0_ concentration ratio vs. reaction time. C_0_ is the initial Orange II dye concentration, and C(t) is the residual concentration at time t. Table 2 shows the removal percentages of the Orange II dye for the series of materials MIL-53, MIL-100, and Fe-BTC as a function of reaction time. As can be seen, Table 2 shows that the highest Orange II dye removal percentage is observed in the 2.5TiO_2_/MIL-100 sample with 96.9%.

#### 2.2.2. Photocatalytic Degradation of Reactive Black Dye

**Figure 9:**
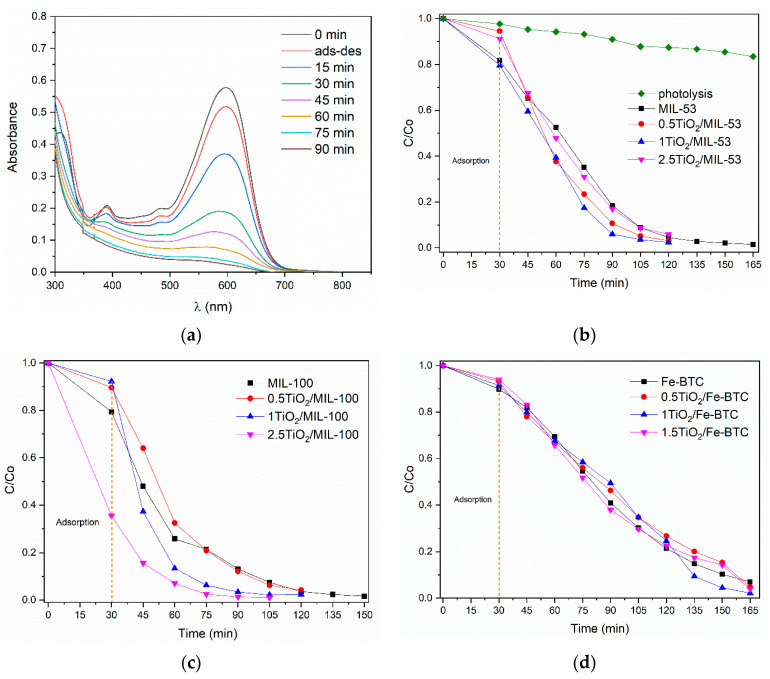
Photocatalytic activity of the Reactive Black 5 dye for the different materials. (**a**) UV–Vis spectra of photocatalytic degradation using MOF 2.5TiO_2_/MIL-100; (**b**–**d**) C(t)/C_0_ concentration ratio vs. reaction time for MIL-53 (Fe), MIL-100 (Fe), and Fe-BTC, respectively.

Figure 9a shows the UV–Vis spectra of the monitoring of the degradation of the Reactive Black dye 5 using MOF 2.5TiO_2_/MIL-100. A strong absorption band is observed at 598 nm corresponding to the N=N chromophore group, being the characteristic peak, and was used to follow the kinetics of the reaction. Table 2 shows the degradation percentages of the Reactive Black 5 dye for the series of materials MIL-53, MIL-100, and Fe-BTC as a function of reaction time. The incorporation of the TiO_2_ NPs into the MOF Fe-BTC slightly improves the degradation percentage of the Reactive Black 5 dye, related to a slight decrease in the bandgap. The comparison of all materials at 105 min of the reaction (Table 2) revealed that the materials of the MIL-100 series showed the highest percentage of dye removal.

The degradation of the Reactive Black 5 dye molecule is complex since its aromatic rings and chromophore group make it very stable (Figure 1). Figure 9b–d show that iron MOFs could degrade the Reactive Black 5 dye in a maximum time of 165 min. Two critical stages of the reaction are observed: The first was at 30 min, corresponding to the adsorption–desorption process of the dye with the photocatalyst surface without the effect of light, and the second stage after the adsorption–desorption equilibrium consisting of the degradation of the RB5 dye. When the light reached the surface of the MOF, it caused the generation of electrons and holes in the valence and conduction band. The incorporation of the titania NPs helped the degradation of the dye to increase; furthermore, with the increase in titania concentration, a greater quantity of colorant degraded in a shorter time. The TiO_2_ allowed for a more efficient separation between the photogenerated electron–hole pairs in the MOF and improved photocatalytic activity.

#### 2.2.3. Kinetics and Mechanism of Dye Degradation

The Langmuir–Hinshelwood kinetic model describes the photocatalytic degradation kinetics, and their expression is as follows:

(1) $$r=-\frac{dC}{dt}=\frac{k_{r}K_{ad}C}{1+K_{ad}C}$$

where k_r_ is the intrinsic reaction constant, and K_ad_, is the adsorption constant at equilibrium. Suppose the adsorption is weak, and the concentration of the organic compound is low. In that case, the K_ad_C factor can be neglected so that the equation is simplified to pseudo-first-order kinetics with an apparent reaction constant K_app_ = k_r_K_ad_. After integrating into the interval [C, C_0_], we obtain:

(2) $$\ln\frac{C}{C_{0}}=K_{app}t$$

Thus, a straight line is obtained when graphing time vs. ln(C/C_0_), where the slope is the apparent reaction constant. Table 2 shows the apparent reaction constants and the correlation coefficients of the degradation of the Orange II and Reactive Black 5 dyes on the materials MIL-53 (Fe), MIL-100 (Fe), and Fe-BTC.

The catalysts with supporting TiO_2_ NPs present an apparent high reaction constant concerning the pristine MOF. The 2.5TiO_2_/MIL-100 catalyst presented a high reaction constant of 0.0453 min^−1^ for Reactive Black 5 and 0.0322 min^−1^ for Orange II. In addition, higher reaction constants were observed in the 2.5TiO_2_/Fe-BTC catalyst for the Orange II dye than for Reactive Black 5, indicating that these materials more effectively degrade Orange II dye.

A possible mechanism for the degradation of dyes could be discussed from the theory of a semiconductor. The irradiation of light in MOFs by photons with energy equal to or greater than the energy of the bandgap excites the electrons (e^−^) of the valence band to the conduction band producing holes (h+) in the valence band. Photogenerated holes (h+) have a strong oxidizing capacity and can act directly on adsorbed organic molecules or react with water molecules to generate radicals (·OH). Hydroxyl radicals (·OH) also have a high oxidation capacity and could rapidly degrade organic molecules adsorbed on the surface. Meanwhile, the photogenerated electrons could be trapped by molecular oxygen to form radicals (·O^2−^), which have a strong oxidative capacity to degrade dyes. The literature mentioned that electron transfer is more efficient if the molecules are pre-adsorbed at the surface with appropriate orientation. Du et al. [33] observed that MIL-53 (Fe) showed low efficiency in the degradation of methylene blue due to the rapid recombination of the charge carriers generated (electron–hole). In our case, we observe a greater adsorption capacity of the dye. The materials are more active, such as those of the MIL-100 (Fe) series.

#### 2.2.4. Stability of the MOFs

Figure S3 shows the TGA curve for the three MOFs used in this investigation. The TGA data reveal that the Fe-BTC is stable up to 300 °C, while for MIL-53 (Fe), it is stable up to 400 and 380 °C for MIL-100 (Fe) according to literature [39,40].

**Figure 10:**
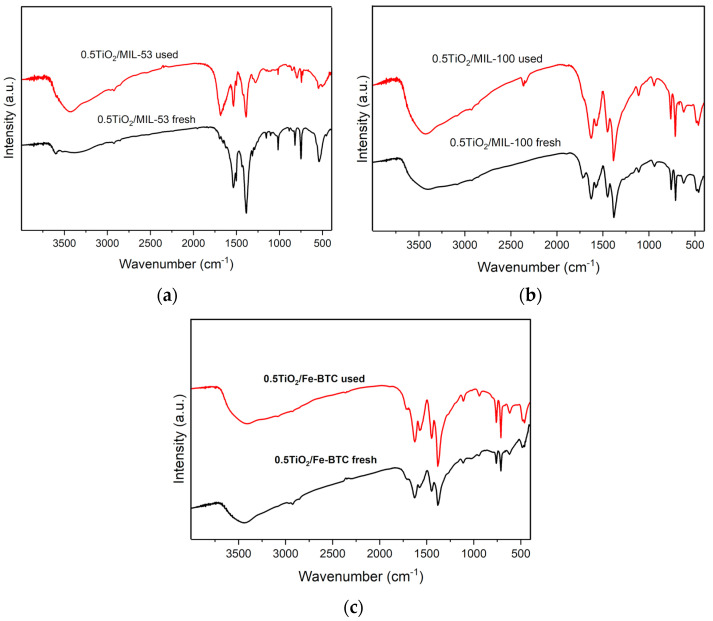
FTIR spectra of the fresh and used composite materials of (**a**) 0.5TiO_2_/Fe–BTC, (**b**) 0.5TiO_2_/MIL–53, and (**c**) 0.5TiO_2_/MIL–100.

Figure 10a shows the FTIR spectra of fresh and used 0.5TiO_2_/MIL-53 in the photocatalytic degradation of Reactive Black 5. As can be observed, FTIR spectrum of fresh 0.5TiO_2_/MIL-53 material shows bands in the zone between 400 and 1750 cm^−1^ corresponding to an organic ligand (therephtalic acid) as well as an interaction between the organic ligand and metallic clusters [39]. However, in the FTIR spectrum of used 0.5TiO_2_/MIL-53 material, the same bands as those of the FTIR spectrum of fresh 0.5TiO_2_/MIL-53 material are not observed, which is indicative of the low stability of this MOF in aqueous medium. In the FTIR spectrum of fresh 0.5TiO_2_/MIL-100 material, bands corresponding to organic linker (BTC), an interaction between metallic clusters and ligand, and characteristics of MOF MIL-100 (Figure 10b) [40] are shown. For example, the bands observed in the zone between 1300 and 1700 cm^−1^ are characteristics of symmetric and asymmetric stretching vibrations of the carboxylate groups in BTC. Other bands were observed at 1000 and 760 cm^−1^, corresponding to C-C vibrational groups and C-H bonds of the aromatic ring of the organic ligand (trimesic acid). Bands at 430, 580, and 635 cm^−1^ attributed to symmetrical and asymmetrical vibrations of the Fe-O bonds were observed. This same bands are observed in the FTIR spectrum of used 0.5TiO_2_/MIL-100 material indicative of the high stability of MOF MIL-100 in aqueous medium.

As the MOF Fe-BTC was synthetized using the same organic ligand as that of the MOF MIL-100, the same bands were observed in the FTIR spectra for both MOFs [36]. In this case, the used 0.5TiO_2_/Fe-BTC material showed the same bands as the fresh material, which demonstrated the high stability of these MOFs in aqueous medium.

#### 2.2.5. Recyclability

**Figure 11:**
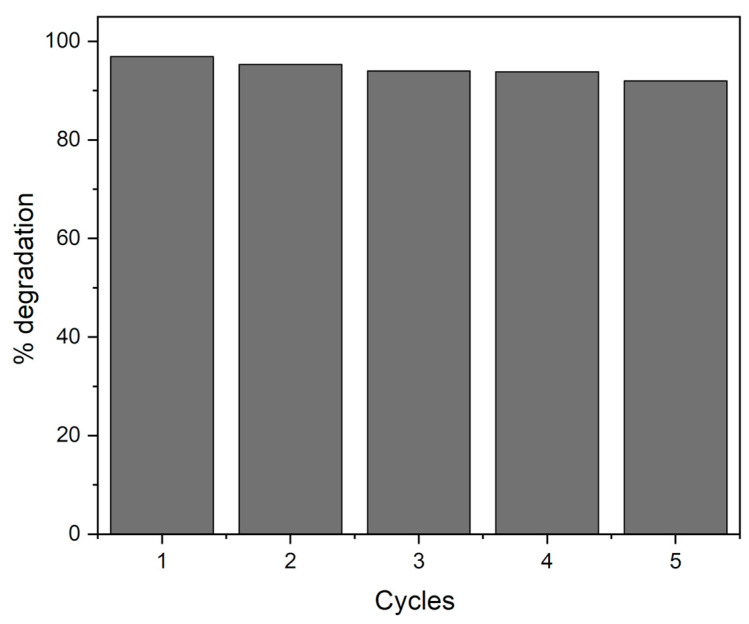
Cycles of reaction for 2.5TiO_2_/MIL-100 composite material in the photocatalytic degradation of Reactive Black 5.

For a material to be applied industrially, it is necessary to determine its recyclability. Figure 11 shows the degradation percentage after several reaction cycles for 2.5TiO_2_/MIL-100 composite material in the photocatalytic degradation of Reactive Black 5. In each of the cycles, the composite material was recovered by centrifugation, subsequently dried, and added to a fresh solution containing the Reactive Black 5 dye. Figure 11 shows a slight decrease in activity after five reaction cycles, showing that these materials can be a good alternative for the removal of anionic dyes in wastewater.

### 2.3. Proposed Mechanism

**Figure 12:**
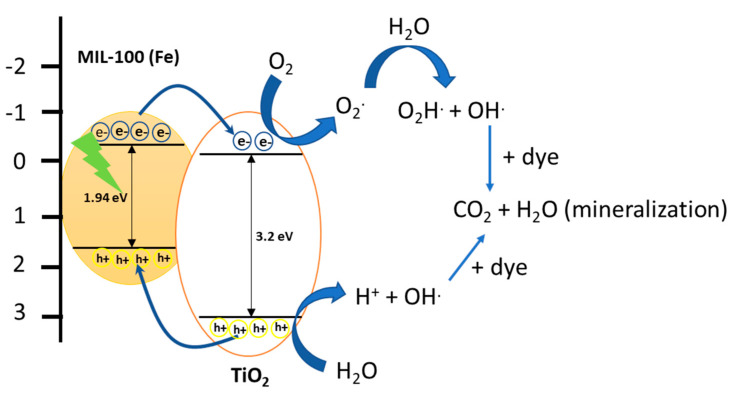
Scheme of a possible reaction mechanism in term of charge transfer in the 0.5TiO_2_/MIL–100 composite material.

Figure 12 shows a possible reaction mechanism in terms of charge transfer. Previously, 2.5TiO_2_/MIL-100 (Fe) composite material showed a band gap of 1.94 eV, indicating that this material can be activated with visible light. When the composite material is irradiated with visible light, the electrons in the valence band pass to the conduction band, leaving a hole in the valence band. The electrons of the conduction band in the MOF are captured by TiO_2_ NPs, which have a lower reduction potential compared with TiO_2_ NPs.

In addition, the transfer of electrons to the conduction band can reduce the recombination of charge carriers, as was observed in the PL results. Once electrons are transferred to the conduction band of TiO_2_, they reduce to O_2_ to form superoxide radicals (O_2_·). In the valence band, the holes (h+) reduce H_2_O-forming H^+^ species and hydroxyl radicals (OH·), these species being responsible for the complete oxidation of the dyes.

## 3. Materials and Methods

### 3.1. Reagents

The deionized water with a conductivity of 0.055 μS used for prepared all solutions was obtained using Millipore DIRECT-Q3 equipment (Millipore Corporation, Bedford, MA, USA). Iron (III) nitrate nonahydrate purity 98%, titanium isopropoxide purity 97%, Reactive Black purity 55%, terephthalic acid purity 98%, trimesic acid purity 95%, sodium hydroxide purity 98%, acetone purity 99.5%, ethanol purity 99.5%, methanol purity 99.9%, acetone purity 99.5%, ethanol purity 99.5%, and methanol purity 99.9% were all purchased from Sigma-Aldrich Merck (St. Louis, MO, USA). Iron (III) chloride hexahydrate 98% purity, hydrochloric acid 38% purity, and hydrogen peroxide solution 30% were obtained from Baker (Ellington, MO, USA). Hydrofluoric acid analytical grade (Reactive Reasol, Mexico City, México) and *N*,*N*-Dimethylformamide purity 99.5% (Merk) were also utilized.

### 3.2. Determination of the Optimum Conditions

The MOF Fe-BTC and Reactive Black 5 dye were used to determine the optimum conditions. Three parameters were analyzed: pH, catalyst mass, and H_2_O_2_ concentration. The photocatalytic reaction was carried out in a glass reactor with a recirculation system to maintain the constant temperature. At the reactor top, an LED lamp (50 W and 50 Hz, laser and LED) (Figure S4) was placed at 18.5 cm. Fe-BTC photocatalyst was added to 25 mL of dye with a 25 mg/L initial concentration. The reaction was stirred for 30 min without light to carry out the adsorption–desorption dye–photocatalyst equilibrium process.

After reaching adsorption–desorption equilibrium, hydrogen peroxide (H_2_O_2_) was added. Subsequently, aliquots were taken at different reaction times (every 15 min until the end of the degradation dye). Then they were filtered through a PTFE Acrodisc with a pore diameter of 0.45 μm. Dye concentrations as a function of reaction time were analyzed on a UV–Vis spectrophotometer (Agilent Technologies, Cary 100 series). Previously, the equipment was calibrated with solutions of different concentrations (5, 10, 25, 50, and 70 mg/L) to determine the residual dye concentration in the samples, following the absorbance at 598 nm for Reactive Black 5 and 485 nm for Orange II.

The experiments were carried out at different pH values (2 to 11), where a 0.1 M NaOH or 0.1 M HCl solution was used to adjust the solution pH. To initiate the photocatalytic degradation process, 10 mg of Fe-BTC photocatalyst was dispersed into the dye solution with 25 mg/L, and 0.5 mL of H_2_O_2_ was added as a sacrificial agent.

**Figure 13:**
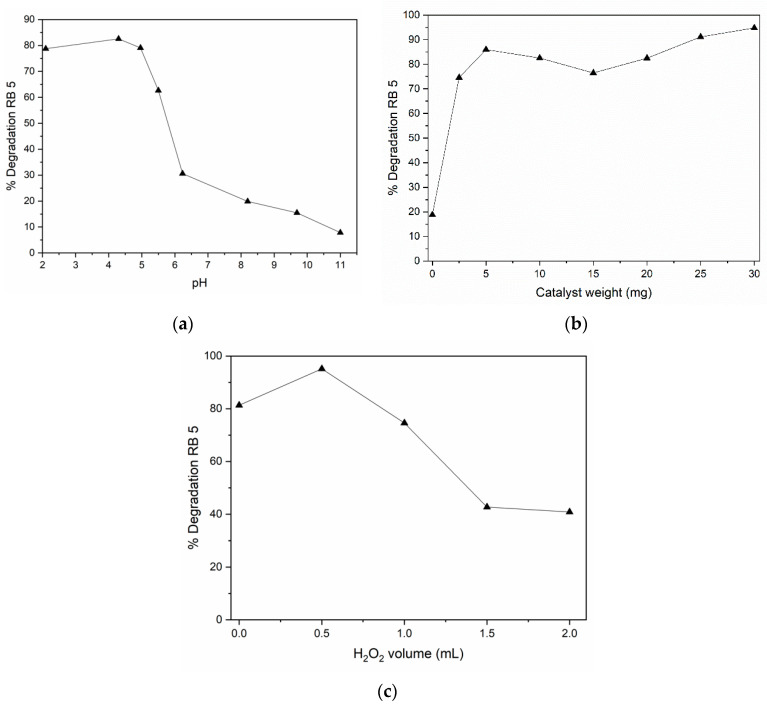
Optimum conditions with varying, (**a**) solution pH, (**b**) catalyst weight, and (**c**) H_2_O_2_ volume.

The effect of pH is one of the most critical parameters for the photocatalytic process since it affects the surface properties of the catalyst [41] (Figure 13a). Photocatalysis is generally favorable under acidic conditions. In Figure 13a, it can be corroborated that the photodegradation is suitable for acidic pH since it is higher at lower pH values, being the optimum at pH = 4.3.

We performed a mass sweep of the Fe-BTC catalyst (0, 2.5, 5, 10, 15, 20, 25, and 30 mg) following the procedure described above using a pH of 4.3 (Figure 13b). The percentage of degradation is strongly dependent on the mass of the catalyst; therefore, tests were carried out with different masses. As can be seen in Figure 13, without the presence of the catalyst and only with light, the degradation of the dye is very low, while when adding the catalyst, it increases considerably, 5 mg being the one that presents the most significant degradation of the Reactive Black 5 dye. However, it is possible to observe that the greater the mass of catalyst, the percentage of degradation decreases. It may be slightly due to light scattering from the presence of a large amount of catalyst. MOFs are materials that adsorb large amounts of dyes since they present high specific surface areas. Therefore, adding more than 25 mg of catalyst makes the adsorption process possibly dominant.

Hydrogen peroxide was varied from 0.5–2 mL and added to the reaction system after 30 min of the adsorption–desorption process. In Figure 13c, it is observed that the addition of H_2_O_2_ improves the photocatalytic activity up to the value of 0.5 mL, decreasing the percentage of degradation by increasing the amount of peroxide of hydrogen. Light promotes oxidation reactions initiated by the presence of free radicals. For these processes to be carried out, oxidizing agents are necessary, allowing these radicals to form. As mentioned, hydrogen peroxide is a powerful non-selective oxidizing agent and an excellent source of free radicals. It is also an ecologically desirable additive since only water and oxygen are generated during decomposition.

### 3.3. Synthesis of the Materials

#### 3.3.1. Fe-BTC Synthesis

A total of 1.76 g of the organic ligand (C_9_O_6_H_6_, trimesic acid) and 3.52 g of nonahydrated iron nitrate (Fe(NO_3_)_3_ • 9H_2_O) were dissolved in 30 mL of DMF (*N*, *N*-dimethylformamide). The before mixture was placed in an ultrasonic bath (Branson 1510) for 5 min. Subsequently, 30 mL of ethanol was added, and it was placed in the ultrasonic bath for five more minutes. Then, 30 mL of deionized water was added, and it was placed in the ultrasonic bath for an additional 30 min. The before mixture was placed in a sand bath at 85 0.5TiO_2_C for 24 h. Afterwards, the solid obtained was filtered and washed with methanol under constant stirring for 24 h. This procedure was repeated three times with fresh methanol. Finally, the solid obtained was dried in an oven at 80 °C for 24 h.

#### 3.3.2. MIL-53 (Fe) Synthesis

A total of 0.57 g of the organic ligand (C_8_O_6_H_6_, terephthalic acid), 0.92g of the hexahydrated iron (III) chloride salt (FeCl_3_ • 6H_2_O), and 0.12 mL of HF was added to 66 mL of DMF, molar ratio 1:1:2:280. The before mixture was placed in a 100 mL autoclave at 150 °C for 72 h. Finally, the solid obtained was filtered and washed with deionized water for a few hours (1 g of MIL-53 in 500 mL of H_2_O). Then, it was dried at room temperature for 24 h [35].

#### 3.3.3. MIL-100 (Fe) Synthesis

A total of 1.66 g of the organic ligand (C_9_O_6_H_6,_ trimesic acid) and 1.94 g of hexahydrated iron (III) chloride salt (FeCl_3_ • 6H_2_O) were added to 60 mL of deionized water [42]. Afterwards, the before mixture was placed in a 100 mL autoclave at 130 °C for 72 h. The solid obtained was filtered and washed with acetone under constant stirring for 24 h. This procedure was repeated three times with fresh acetone. Finally, it was dried in atmospheric air.

#### 3.3.4. Incorporation of TiO_2_ NPs into MOF

The materials supported were synthesized using the solvothermal method. In this case, the same methodology outlined in Section 3.3.1, Section 3.3.2 and Section 3.3.3 was used to synthesize the supporting materials. However, when the iron metal salt and the organic ligand (terephthalic acid or trimesic acid) were dissolved entirely during continuous stirring, the exact amount corresponding to the required percentage (0.5, 1.0, and 2.5% wt.) of TiO_2_ was added.

### 3.4. Characterization of Materials

The materials were characterized with X-ray diffraction (XRD) in an X’Pert diffractometer (Philips, Almelo, The Netherlands) using a CuKα (λ = 1.54 Å) radiation source, with a step size of 0.02° in 2θ per second in the range of 5 to 50° in 2θ, 45 kV, and 40 mA. Fourier transform infrared spectra (FTIR) were determined with the KBr pellet technique, using controlled amounts of KBr (ratio of 1 mg sample to 100 mg KBr) in a Magna-IR 750 device (Thermo Nicolet, Champaign, IL, USA). Raman spectra were recorded with an inVia microscope (Renishaw, Gloucestershire, UK), using a green laser (λ = 532 nm) as the excitation line, 1% laser power, and a measurement range from 100 to 2000 cm^−1^. A UV–Vis spectrometer (Agilent Technologies Cary 100 series, Mulgrave, Australia) was used to measure the degradation of the dye (bandgap). The photoluminescence (PL) was recorded using a Hitachi F-7000 with the exciting wavelength at 400 nm.

### 3.5. Photocatalytic Test

For all the photocatalytic tests with different synthesized materials, 5 mg of photocatalyst was dispersed in 25 mL of dye with a 25 mg/L initial concentration at pH 4.3. After 30 min of the adsorption–desorption process, 0.5 mL of H_2_O_2_ was added, and the solution was irradiated with an LED lamp of 50 W and 50 Hz (light and LED) in the visible region (Figure S4). Subsequently, aliquots were taken at different reaction times (every 15 min until the end of the degradation of the dye). These were filtered through a Millipore Millex-LCR Hydrophilic membrane with a pore diameter of 0.45 μm. Residual dye concentrations in the samples taken as a function of reaction time were analyzed on a UV–Vis spectrophotometer (Agilent Technologies, Cary 100 series).

## 4. Conclusions

MOF series were successfully synthesized using the solvothermal method. The incorporation of the TiO_2_ NPs did not affect the crystal structure of the MOFs. The bandgap of the MOFs was slightly reduced with the incorporation of the TiO_2_ NPs, thus requiring less energy to activate them. In addition, all the materials showed that they could be activated in the visible region. The MIL-53 (Fe) and MIL-100 (Fe) series showed better results for the photocatalytic degradation of the Reactive Black 5 and Orange II dyes. The activation of the MOF with visible light allows the valence band’s electrons to migrate to the conduction band, where these electrons migrate to the conduction band of TiO_2_, thus decreasing the recombination of the charge carriers in the MOF coupled with an increase in photocatalytic activity. MIL-100 and Fe-BTC composite materials appeared very stable in the aqueous medium. The synthesized materials could therefore be excellent candidates for the degradation of colorants in contaminated water.

## Figures and Tables

**Table 1:** The bandgap of all the materials (determined by Tauc equation).

Sample	Bandgap (eV)	
	Direct	Indirect
MIL-53 (Fe)	2.45	2.9
0.5TiO_2_/MIL-53	2.42	2.9
1TiO_2_/MIL-53	2.41	2.8
2.5TiO_2_/MIL-53	2.43	2.9
MIL-100 (Fe)	1.95	2.72
0.5TiO_2_/MIL-100	1.96	2.72
1TiO_2_/MIL-100	1.94	2.72
2.5TiO_2_/MIL-100	1.94	2.72
Fe-BTC	2.45	3.1
0.5TiO_2_/Fe-BTC	2.42	3.1
1TiO_2_/Fe-BTC	1.7	2.65
2.5TiO_2_/Fe-BTC	2.45	3.09

**Table 2:** Percentage of degradation at 105 min and apparent reaction constant for all samples.

Sample	% Degradation		Apparent Reaction Constant (min^−1^)			
	Orange II	RB 5	Orange II	R^2^	RB 5	R^2^
MIL-53	95.2	91.2	0.0262	0.8892	0.0236	0.9073
0.5TiO_2_/MIL-53	93.4	94.9	0.0247	0.8654	0.0245	0.8653
1.0TiO_2_/MIL-53	95.9	96.4	0.0292	0.8673	0.0279	0.8723
2.5TiO_2_/MIL-53	95.4	91.0	0.0258	0.8864	0.0199	0.8621
MIL-100	94.0	92.5	0.0236	0.8674	0.255	0.9565
0.5TiO_2_/MIL-100	91.3	93.7	0.0244	0.8652	0.0234	0.8964
1.0TiO_2_/MIL-100	94.3	97.7	0.0270	0.9341	0.0335	0.9234
2.5TiO_2_/MIL-100	96.9	98.9	0.0322	0.8865	0.0453	0.9856
Fe-BTC	91.2	69.8	0.0223	0.9542	0.0133	0.8865
0.5TiO_2_/Fe-BTC	-	65.3	-	-	0.0125	0.8123
1.0TiO_2_/Fe-BTC	-	65.2	-	-	0.0161	0.7432
2.5TiO_2_/Fe-BTC	92.3	70.3	0.0243	0.9453	0.0135	0.8221

## Data Availability

Not applicable.

## Supplementary Materials

The following supporting information can be downloaded at: https://www.mdpi.com/article/10.3390/molecules27207078/s1. It includes the power X-ray diffraction pattern of nickel oxide nanoparticles, Figure S1; Method for the determination of direct and indirect bandgap, Figure S2; Plots of TGA of MOFs pristine, Figure S3; Plot of Emission spectrum of LEDs lamp used in the experiments photocatalytic, Figure S4.
