# Supplementary material for: Photocatalytic Degradation of Dyes Using Titania Nanoparticles Supported in Metal-Organic Materials Based on Iron
Source: Molecules. 2022 Oct 20;27(20):7078. doi: 10.3390/molecules27207078 (PMC9610564; doi:10.3390/molecules27207078)
Supplement: Supplementary file 1 [file molecules-27-07078-s001.zip › molecules-1932654-supplementary.pdf]

Supplementary information

Tables and Figures

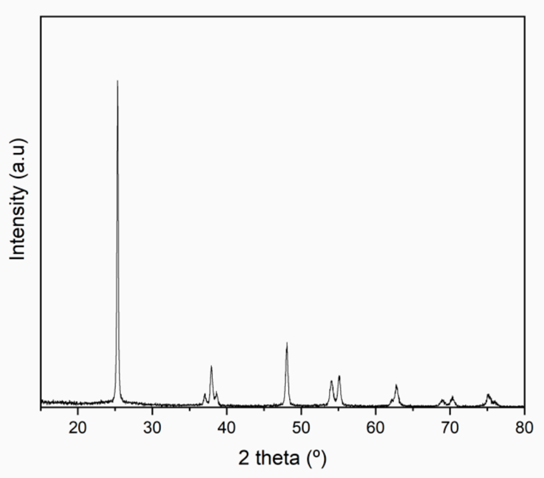

Figure S1. Powder X-ray diffraction pattern of synthesized TiO_2 NPs.

The diffuse reflectance spectrum is converted to the Kubelka Munk function F(R), equivalent to the absorption coefficient. The Tauc model's α symbol is replaced with F(R), and this is expressed according to the following equation:

$$(F(R) * h\nu) = A(h\nu - E_g)^n$$

In addition, for indirect transitions, phonon energy is involved in the network (E_Ω). once added to the model, the positive sign is related to the emission of a phonon and the negative to phonon absorption.

$$(F(R)h\nu) = A\,(h\nu - E_g \pm E_\Omega)^n$$

Drawing the Kubelka-Munk function $(F(R)h\nu)^n$ versus hν, where, for a direct transition with n = 1/2 and an indirect transition with n = 2 (**Figure S1**). Finally, the interval Eg can be obtained with a line tangent to the point of inflection of the curve of the graph $(F(R)h\nu)^n$ against $h\nu$, where $h\nu$ = 0 .

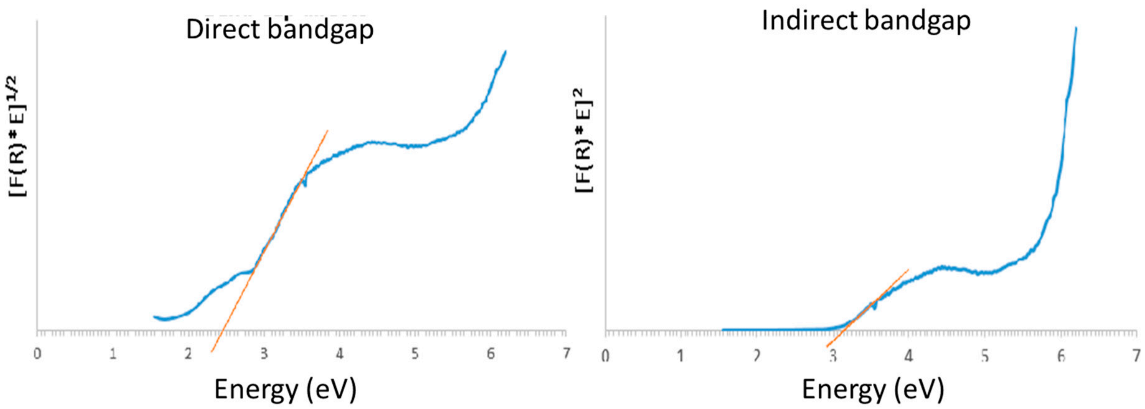

Figure S2. Graph for the determination of direct and indirect bandgap in the MIL-100 sample.

The TGA curve of Fe-BTC shows three process main between room temperature and 600 ºC. The first on (approx 6.5%), in a range from 25 to 150 ° C is attributed to the desorption of the free water molecules inside the pores, and most likely, these water molecules interact with the iron trimers. The second one, the descomposition of organic ligand (Benzene-1,3,5-tricarboxylic acid) ocurrs in two steps in a range from 245 to 410 ºC with weight loss of approx. 40 % . Finally, the last process above 500ºC, Fe-BTC is transformed into amorphous Fe_2O_3 (Figure S3).

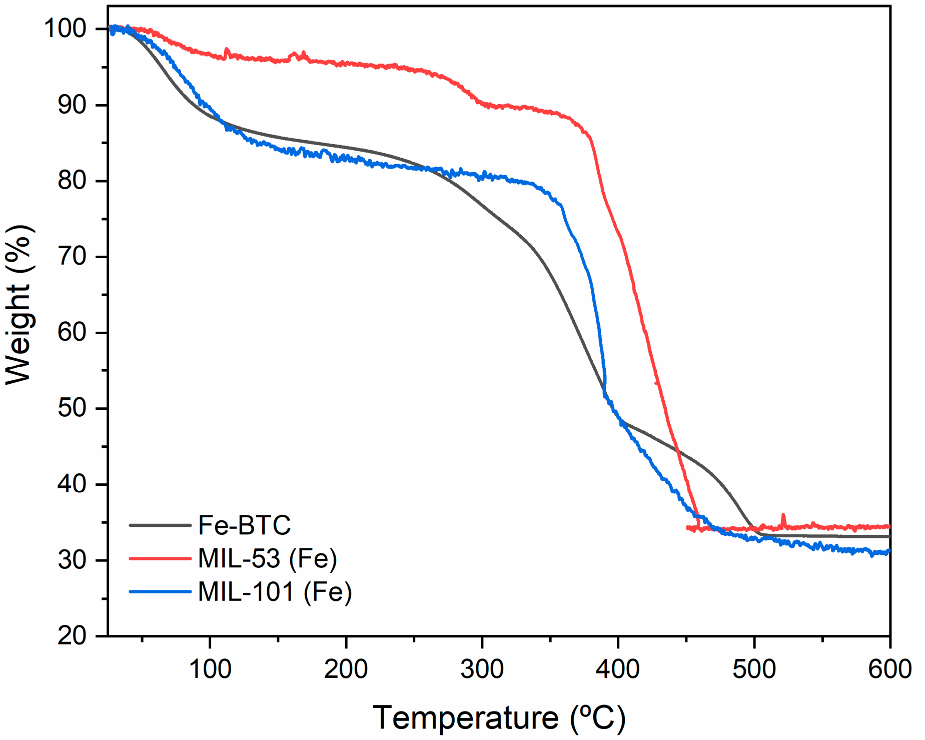

Figure S3. TGA of the MOFs pristine

Figure S3 shows the emission spectrum of the LEDs lamp used in the photocatalytic activity. As can be seen, two peaks emission are observed at 568 and 455 nm, which are within the region of the electromagnetic spectrum of visible light (350–750 nm).

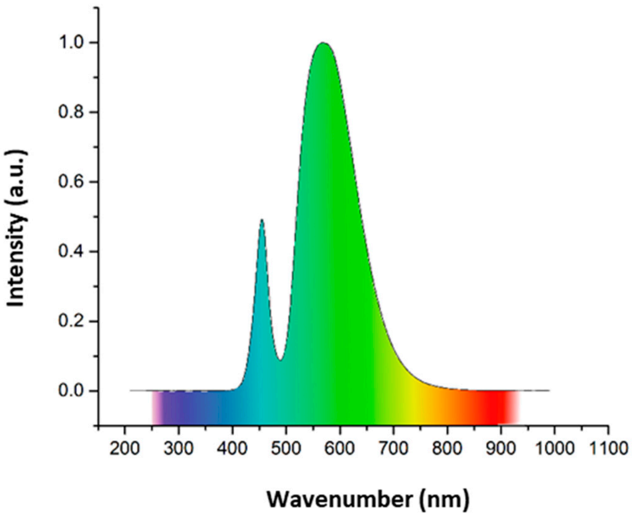

Figure S4. Emission spectrum of LEDs lamp used in the experiments.
